# Treatment patterns and survival in metastatic castration‐sensitive prostate cancer in the US Veterans Health Administration

**DOI:** 10.1002/cam4.4372

**Published:** 2021-11-02

**Authors:** Stephen J. Freedland, Rickard Sandin, Janvi Sah, Birol Emir, Qiao Mu, Anna Ratiu, Agnes Hong, Lucile Serfass, Scott T. Tagawa

**Affiliations:** ^1^ Division of Urology Department of Surgery Cedars‐Sinai Medical Center Los Angeles California USA; ^2^ Veterans Affairs Health Care System Durham North Carolina USA; ^3^ Pfizer AB Sollentuna Sweden; ^4^ STATinMED Research Ann Arbor Michigan USA; ^5^ Pfizer Inc. New York New York USA; ^6^ Astellas Inc. Northbrook Illinois USA; ^7^ Pfizer Inc. Paris France; ^8^ Division of Hematology & Medical Oncology and Department of Urology Weill Cornell Medicine New York Presbyterian Hospital New York New York USA

**Keywords:** anti‐androgen, LHRH agonist, LHRH antagonist, overall survival, prostate cancer

## Abstract

**Background:**

Limited real‐world data exist on treatment patterns and outcomes in patients with metastatic castration‐sensitive prostate cancer (mCSPC).

**Methods:**

A retrospective cohort study was conducted, using the Veterans Health Administration claims database (April 2013–March 2018). Among 369,734 prostate cancer patients, we selected all men who developed metastases within 90 days before or after medical/surgical castration and who received androgen deprivation therapy (ADT). Patients were categorized into four cohorts: ADT‐only (± <90‐day nonsteroidal anti‐androgen [NSAA] use), ADT + NSAA, ADT + docetaxel, and ADT + abiraterone. Main outcomes were treatment patterns, time‐to‐progression to metastatic castration‐resistant disease, and overall survival. Multivariable analysis and sensitivity analysis were conducted.

**Results:**

Of 1395 patients, 874 (63%) received ADT‐only, 338 (24%) received ADT + NSAA, 108 (8%) received ADT + docetaxel, and 75 (5%) received ADT + abiraterone. Proportions on ADT‐only and ADT + NSAA declined (from 66% to 60% and from 31% to 17%, respectively) over the study period, while proportions prescribed ADT + docetaxel or abiraterone increased from 3% to 9% and from 1% to 15%, respectively. Patients treated with ADT + NSAA had similar risks of castration‐resistant disease (hazard ratio [HR] 1.05; 95% confidence interval [CI]: 0.87, 1.26) and overall mortality (HR 1.22; 95% CI: 0.97, 1.54) as ADT‐only.

**Conclusions:**

Most patients with mCSPC initiating ADT received ADT‐only or ADT + NSAA, despite the emergence of docetaxel and novel hormonal therapies. Even in the most recent period (2017 to early 2018), only 24% of men received intensified therapy with agents known to prolong survival versus ADT‐only. These data in real‐world clinical practice suggest substantial room for improved outcomes in patients with mCSPC.

## Graphical abstract

A retrospective cohort study of 1395 men with metastatic castration‐sensitive prostate cancer initiating androgen deprivation therapy (ADT) found that the vast majority received ADT alone or with a first‐generation anti‐androgen. Despite the emergence of life‐prolonging treatments such as docetaxel and novel hormonal therapies, only a small proportion of patients received these treatments, indicating substantial room for improved outcomes in this population.

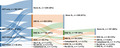

**Lay summary**

This study looked at the treatment approaches and survival outcomes for patients with metastatic castration‐sensitive prostate cancer (mCSPC) in a real‐world setting. Of 1395 men with mCSPC initiating androgen deprivation therapy (ADT), 87% of patients received ADT alone or with a first‐generation anti‐androgen. The risk of developing castration‐resistant prostate cancer and overall survival were similar with the two approaches. Despite the emergence of life‐prolonging treatments such as docetaxel and novel hormonal therapies, only a small share of patients received these treatments, indicating significant room for improved outcomes among patients with mCSPC.

## 1 INTRODUCTION

Prostate cancer is the most common cancer diagnosis and the second most common cause of cancer‐related mortality in men in the United States, with an estimated 248,530 new cases and 34,130 deaths in 2021.^1^ Metastatic castration‐sensitive (i.e., non‐castrate) prostate cancer (mCSPC) is prostate cancer that has extended beyond the prostate in the setting of normal serum levels of testosterone, that usually responds to androgen deprivation therapy (ADT).

Current practice guidelines recommend ADT with novel hormonal therapies (NHTs: abiraterone, apalutamide, or enzalutamide) or docetaxel as the initial standard of care for patients with mCSPC.^2^, ^3^, ^4^ Short‐term use of a first‐generation nonsteroidal anti‐androgen (NSAA [bicalutamide, flutamide, or nilutamide]) is currently recommended for treating temporary testosterone flare commonly seen with many ADT approaches.^4^ An alternative approach is to use ADT in conjunction with an NSAA to achieve complete androgen blockade, although current guidelines note that there is likely minimal added benefit of combined androgen blockade.^3^

In recent years, multiple clinical trials have demonstrated that adding chemotherapy or NHTs to ADT leads to significantly prolonged progression‐free survival (PFS) and overall survival (OS) in patients with mCSPC.^5^, ^6^, ^7^, ^8^, ^9^, ^10^, ^11^, ^12^, ^13^, ^14^, ^15^ Based on these findings, a combination of ADT and docetaxel, abiraterone, apalutamide, or enzalutamide is recommended for mCSPC.^2^, ^3^

While data on survival rates are accumulating from clinical trials, limited real‐world evidence of clinical benefit exists for patients with mCSPC in the United States.^16^, ^17^, ^18^ Real‐world data can validate and expand on trial outcomes as well as provide information on treatment patterns and uptake of new pharmacotherapies across a broader patient population. The objective of this study was to evaluate patient characteristics, treatment patterns, time‐to‐progression to metastatic castration‐resistant prostate cancer (mCRPC), and OS among men with mCSPC who initiated either ADT‐only or ADT with NSAA, docetaxel, or abiraterone acetate, using the US Veterans Health Administration (VHA) database.

## 2 METHODS

### 2.1 Patient Selection and Study Design

This retrospective cohort study was based on claims data extracted from the VHA database within a 5‐year study period (1 April 2013–31 March 2018), which comprised a 4‐year identification period (1 April 2014–31 March 2018) and a 12‐month pre‐index (baseline) period. From a cohort of 369,734 patients with a first claim for prostate cancer during the study period, 1395 had metastases within 90 days before or after medical/surgical castration (the initial ADT date) during the identification period and received ADT‐only or ADT with the addition of NSAA, docetaxel, or abiraterone. CSPC was defined as use of ADT for ≥3 months (without a gap of ≥90 days between two claims), and without documented use of NSAA, NHTs, or docetaxel in the 12 months preceding the initial ADT date. Patients who received antineoplastic therapies during the baseline period were excluded, as were patients receiving enzalutamide, given that the study time period pre‐dated its approval for mCSPC (complete selection criteria are shown in Figure S1). Prior adjuvant ADT was allowed, that is, if received prior to 12 months before the date of first metastases. Patients with mCSPC who met the selection criteria were categorized into four cohorts:

- **ADT + abiraterone (*n* = 75)**: Patients with claims for abiraterone within 4 months after or within 30 days prior to ADT initiation were classified as ADT + abiraterone.
- **ADT + docetaxel (*n* = 108)**: Patients with claims for docetaxel within 4 months after or within 30 days prior to ADT initiation were classified as ADT + docetaxel.
- **ADT + NSAA (*n* = 338):** Patients prescribed NSAAs for ≥3 months after the initial ADT date were classified as ADT + NSAA. Patients treated with NSAA for <3 months (likely for testosterone flare) were classified as ADT‐only.
- **ADT‐only (*n* = 874):** Patients with mCSPC who received ADT‐only (i.e., did not receive the treatments noted above in conjunction with ADT or were treated with NSAA for <3 months) were classified as ADT‐only.

The index date was defined as the start of ADT, abiraterone, docetaxel, or NSAA, whichever occurred first.

### 2.2 Study measures

#### 2.2.1 Baseline patient characteristics

Baseline demographics (age, race, and index year) and clinical variables (comorbidity index score,^19^ which did not include cancer, and individual comorbidities) were measured during the 12 months prior to the index date. Time from diagnosis of metastasis to index date plus site of metastasis (lymph node, respiratory and digestive, other [including bone], and unspecified sites) were measured within ±90 days of the index date. Prognostic factors, including serum prostate‐specific antigen (PSA), hemoglobin, and alkaline phosphatase concentrations were measured within 6 months prior to the index date to include the maximum number of patients with the selected prognostic variables. When multiple values were available, we used the values closest to, but before, the index date.

#### 2.2.2 Survival outcomes

Patients were followed from the index date to the earliest of death or the end of the study. OS and progression to mCRPC were evaluated during follow‐up. Progression to mCRPC was defined as:

- Change in index therapy or evidence of cabazitaxel, mitoxantrone, abiraterone, docetaxel, enzalutamide, radium‐223, or sipuleucel‐T after 4 months from the index date until the end of follow‐up; or
- An increase in PSA concentration of ≥25% and 2 ng/ml from the nadir value (lowest PSA value ≥14 days from the index date) evaluated from the index date until discontinuation of ADT (defined as a gap >90 days between 2 ADT claims); or
- Among patients who re‐initiated ADT, if their first PSA value after re‐initiation was ≥the last PSA value before re‐initiation of ADT.

Time‐to‐event endpoints were determined from the index date. Given low statistical power, we did not evaluate time to mCRPC and OS in ADT + abiraterone‐ or ADT + docetaxel‐treated patients.

#### 2.2.3 Treatment patterns

Patients who progressed to mCRPC were evaluated for subsequent lines of treatment. Among patients who progressed to mCRPC, evidence of change in therapy (cabazitaxel, mitoxantrone, abiraterone acetate, docetaxel, enzalutamide, radium‐223, or sipuleucel‐T) observed on or after the mCRPC date was considered the beginning of second‐line (2L) therapy. Additionally, if patients in the ADT + abiraterone group or ADT + docetaxel group reinitiated abiraterone or docetaxel, respectively (after a lapse of >90 days) during follow‐up, it was considered 2L treatment. Change in therapy after 2L treatment was defined as third‐line (3L) treatment, and change in therapy after the 3L treatment was defined as fourth‐line treatment.

### 2.3 Statistical analysis

Descriptive statistics were generated for all study variables. This included means and standard deviations for normally distributed continuous variables, and medians and interquartile ranges for skewed continuous variables. Treatment patterns and distribution across lines of therapy and sequencing were analyzed with Sankey flow diagrams connecting a series of events over time to visualize treatment along the mCSPC patient journey. The Kaplan–Meier analysis was used to estimate the unadjusted survival curves for mCRPC and OS.

Multivariable analysis with inverse probability treatment weighting (IPTW) was applied to the main analysis to balance patient characteristics and potential confounders when comparing outcomes among ADT + NSAA and ADT‐only cohorts while retaining all eligible subjects. Lasso penalized regression was used to select covariates with the most explanatory value for the survival outcomes.^20^ Covariates (listed in Table 1) were used to define the probability of a patient receiving a certain treatment.^21^ Age and race were exact matched in IPTW. Post‐IPTW, Schoenfeld residuals plots indicated that body mass index (BMI) produced a nonlinear effect on the models, and hence BMI and BMI^2 (quadratic term) were included in the survival models to offset non‐proportionality assumption (model fit). Additionally, site of metastatic diagnosis, time from metastatic diagnosis to index date, and log PSA concentration were included in the model based on clinical rationale. Progression to mCRPC and OS were evaluated using a Cox proportional hazard model while adjusting for the above covariates.

**TABLE 1:** Baseline demographic and clinical characteristics among patients with mCSPC

	ADT‐only (reference)	ADT + NSAA	STD^a^	ADT + abiraterone	STD^a^	ADT + docetaxel	STD^a^
Sample size (N = 1395); n (%)	874 (63)	338 (24)		75 (5)		108 (8)	
Age (y), n (%)							
Mean (SD)	73.4 (9.8)	74.5 (9.7)	11.09	75.3 (8.6)	20.50	65.8 (7.1)	89.18
Median (Q25–Q75)	72 (67–82)	74 (67–82)		73 (70–83)		67 (61–71)	
≤59	60 (7)	17 (5)	7.76	2 (3)	19.76	20 (19)	35.43
60–69	297 (34)	110 (33)	3.05	15 (20)	31.79	55 (51)	34.70
70–79	251 (29)	88 (26)	6.01	33 (44)	32.05	30 (28)	2.08
≥80	266 (30)	123 (36)	12.64	25 (33)	6.20	3 (3)	79.97
Race, n (%)							
White	555 (64)	238 (70)	14.72	49 (65)	3.81	78 (72)	18.71
Black	247 (28)	77 (23)	12.58	18 (24)	9.68	23 (21)	16.15
Other/unknown	72 (8)	23 (7)	5.43	8 (11)	8.28	7 (6)	6.71
NCI comorbidity index score							
Mean (SD)	1.5 (1.7)	1.4 (1.7)	6.00	1.4 (1.6)	7.4	1.1 (1.5)	30.0
Median (Q25–Q75)	1 (0–2)	1 (0–2)		1 (0–2)		1 (0–1)	
Baseline comorbidities, n (%)							
Acute coronary syndrome	61 (7)	17 (5)	8.21	3 (4)	13.07	5 (5)	10.04
Angina pectoris	18 (2)	12 (4)	9.03	3 (4)	11.29	1 (1)	9.34
Arrhythmia	63 (7)	33 (10)	9.17	2 (3)	21.03	3 (3)	20.41
Chronic obstructive pulmonary disease	135 (15)	60 (18)	6.19	6 (8)	23.24	14 (13)	7.10
Congestive heart failure	71 (8)	34 (10)	6.73	5 (7)	5.55	2 (2)	29.07
Diabetes	298 (34)	94 (28)	13.61	26 (35)	1.20	31 (29)	11.61
Hyperlipidemia	470 (54)	188 (56)	3.70	36 (48)	11.53	54 (50)	7.54
Hypertension	645 (74)	245 (72)	2.96	49 (65)	18.40	66 (61)	27.25
Myocardial infarction	26 (3)	16 (5)	9.14	4 (5)	11.79	1 (1)	14.83
Stroke	50 (6)	27 (8)	8.97	3 (4)	7.98	6 (6)	0.71
Urinary tract infection	101 (12)	30 (9)	8.85	9 (12)	1.37	10 (9)	7.51
Obesity	297 (34)	96 (28)	12.05	21 (28)	12.92	41 (38)	8.28
Prognostic variables^b^							
PSA value (ng/mL)							
Mean (SD)	256.5 (696.1)	303.1 (773.9)	6.33	238.3 (524.3)	2.96	338.1 (866.8)	10.38
Median (Q25–Q75)	31.5 (8.5–126.2)	51.3 (13.0–207.2)		36.4 (11.1–96.1)		71.6 (16.3–290.4)	
Hemoglobin (g/dL)							
Mean (SD)	12.8 (2.1)	13.1 (2.0)	13.57	12.1 (2.1)	31.58	13.4 (2.0)	29.37
Median (Q25–Q75)	13.0 (11.6–14.2)	13.2 (12.1–14.4)		12.4 (10.6–13.6)		13.7 (12.3–14.7)	
Alkaline phosphatase (IU/L)							
Mean (SD)	194.3 (250.0)	237.3 (328.9)	14.75	216.4 (281.1)	8.31	267.2 (313.2)	25.74
Median (Q25–Q75)	102.0 (75.0–252.7)	112.0 (77.0–283.0)		116.0 (78.0–265.0)		140.0 (84.5–350.1)	
Site of metastasis,^c^ n (%)							
Lymph nodes	210 (24)	59 (17)	16.25	26 (35)	23.43	25 (23)	2.07
Respiratory and digestive	48 (5)	20 (6)	1.83	3 (4)	7.00	18 (17)	36.05
Other sites (including bone)	639 (73)	282 (83)	25.20	60 (80)	16.26	96 (89)	40.98
Unspecified	124 (14)	40 (12)	6.99	6 (8)	19.74	11 (10)	12.23

Abbreviations: ADT, androgen deprivation therapy; mCSPC, metastatic castration‐sensitive prostate cancer; NCI, National Cancer Institute; NSAA, nonsteroidal anti‐androgen; PSA, prostate‐specific antigen; SD, standard deviation; STD, standardized difference.

^a^ STD: standardized difference=100* (actual STD) measured versus ADT‐only. A standardized difference >10 is considered significant.

^b^ Prognostic variables were evaluated within 6 months prior to the index date.

^c^ Metastatic‐related variables were evaluated within ±90 days from the index date.

#### 2.3.1 Sensitivity analysis

To confirm the robustness of the multivariable analysis, 1:2 propensity score matching (PSM) was used as sensitivity analysis. The same covariates as in the main analysis were adopted to define the propensity scores for the patients with mCSPC. Each patient in the ADT + NSAA cohort was matched to two patients in the ADT‐only cohort with the closest propensity score. The “nearest neighbor” method without replacement and with a caliper of 0.01 was used to select the matched samples. The balance of covariates between treatment groups was determined using the absolute standardized difference of the mean ≤0.10.

## 3 RESULTS

Based on the predefined selection criteria, a total of 1395 cases of mCSPC were identified in the VHA database including 874 (63%) ADT‐only, 338 (24%) ADT + NSAA, 75 (5%) ADT + abiraterone, and 108 (8%) ADT + docetaxel patients (Table 1). Of the patients who had ADT + NSAA, 98% received bicalutamide.

Compared with the ADT‐only cohort, ADT + docetaxel patients were younger and had fewer comorbidities, but had greater disease burden in terms of higher PSA and metastases (respiratory and digestive, and other sites including bone), while ADT + abiraterone patients were older, generally had fewer cardiovascular comorbidities, and had higher disease burden in terms of PSA and metastases (other sites including bone); there was also a higher proportion of patients with metastatic lymph node involvement in this group. Patients on ADT + NSAA had similar comorbidity to the ADT‐only cohort but were older, with higher disease burden in terms of higher PSA levels and more metastatic sites. Among men with visceral metastases, the majority received ADT alone (54%). ADT + NSAA and ADT + docetaxel patients were less likely to be Black compared to ADT‐only patients (Table 1). Descriptive statistics (and unadjusted for baseline characteristics) on treatment by race are shown in Table S1.

**FIGURE 1:**
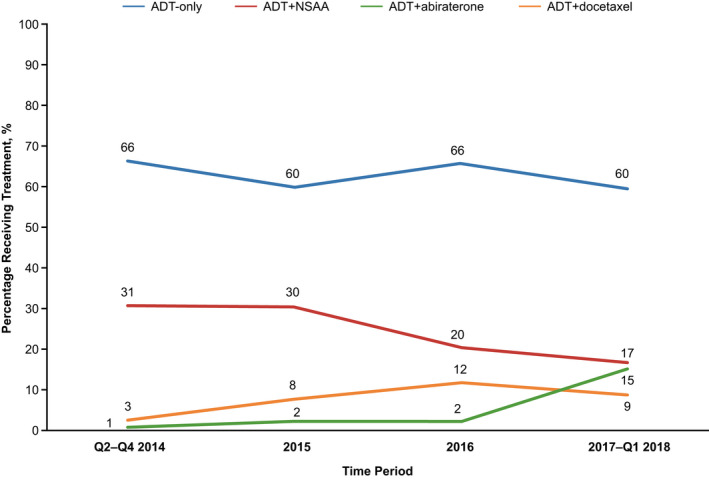
Treatment trends over time among patients with mCSPC

From Q2 2014 to Q1 2018, the number of patients receiving ADT‐only (66%–60%) and ADT + NSAA (31%–17%) declined, whereas the number of patients prescribed ADT + docetaxel (3%–9%) and ADT + abiraterone (1%–15%) increased (Figure 1). In the most recent period of 2017–Q1 2018, 24% of patients received either docetaxel or abiraterone along with ADT.

### 3.1 Survival outcomes

#### 3.1.1 Unadjusted analysis

**FIGURE 2:**
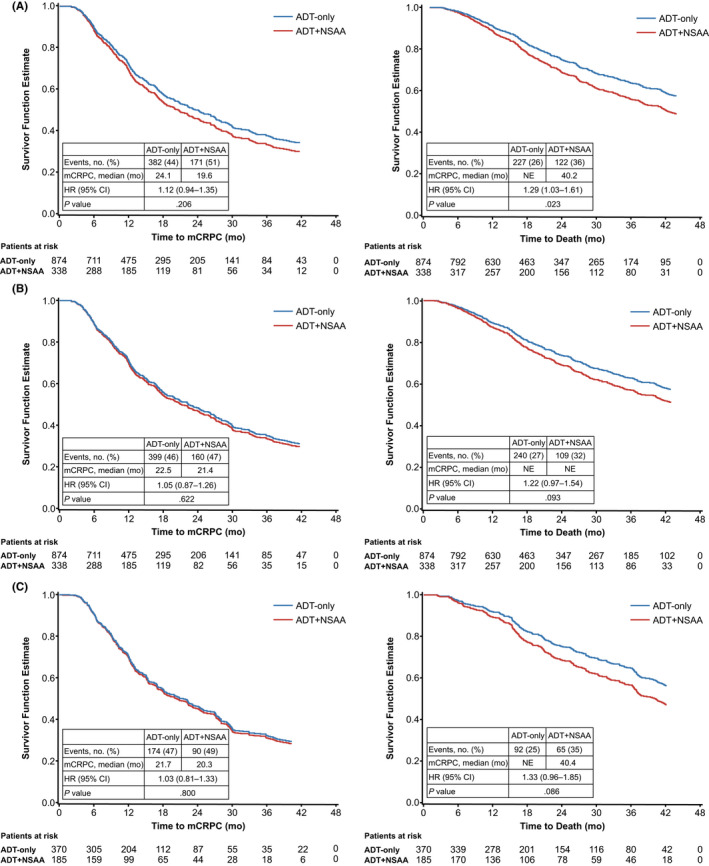
(A) Unadjusted time to mCRPC and OS among patients with mCSPC treated with ADT + NSAA versus ADT‐only; (B) post‐IPTW adjusted time to mCRPC and OS among patients with mCSPC treated with ADT + NSAA versus ADT‐only; and (C) sensitivity analysis – post‐PSM adjusted time to mCRPC and OS among patients with mCSPC treated with ADT + NSAA versus ADT‐only

The median follow‐up was 49.3 months for patients treated with ADT‐only, 53.6 months for ADT + NSAA, 19.1 months for ADT + abiraterone, and 45.3 months for ADT + docetaxel. Compared with the ADT‐only cohort, with a median time to mCRPC of 24.1 months, unadjusted time to mCRPC was similar for ADT + NSAA (hazard ratio [HR]: 1.12 [95% confidence interval (CI): 0.94, 1.35]; time to mCRPC: 19.6 months; Figure 2A). Compared with the ADT‐only cohort, unadjusted OS was lower for the ADT + NSAA cohort (HR: 1.29 [95% CI: 1.03, 1.61]; Figure 2A).

### 3.2 Multivariable adjusted analysis

Multivariable analysis was restricted to a comparison of ADT + NSAA with ADT‐only. Table S2 lists the post‐IPTW and post‐PSM baseline demographic and clinical characteristics. After applying IPTW, all patient characteristics were balanced; median follow‐up was 52.2 months for ADT‐only and 50.1 months for ADT + NSAA. Forty‐seven percent of patients treated with ADT + NSAA progressed to mCRPC compared with 46% of ADT‐only patients. Patients treated with ADT + NSAA exhibited similar risk of progression to mCRPC (HR: 1.05 [95% CI: 0.87, 1.26]), with a median time to mCRPC of 21.4 versus 22.5 months compared to ADT‐only. During the entire follow‐up period, 32% of ADT + NSAA patients died compared with 27% of the ADT‐only cohort. Patients treated with ADT + NSAA had a similar risk of death (HR: 1.22 [95% CI: 0.97, 1.54]) compared to ADT‐only, with median OS not reached in either cohort (Figure 2B).

### 3.3 Sensitivity analysis

Following PSM, 370 ADT‐only and 185 ADT + NSAA pairs were matched. Results of the PSM sensitivity analysis for progression to mCRPC and OS were consistent with those of the IPTW analysis, confirming no difference in time to mCRPC (HR: 1.03 [95% CI: 0.81, 1.33]) and risk of death (HR: 1.33 [95% CI: 0.96, 1.85]) between ADT + NSAA and ADT‐only (Figure 2C).

### 3.4 Treatment patterns

**FIGURE 3:**
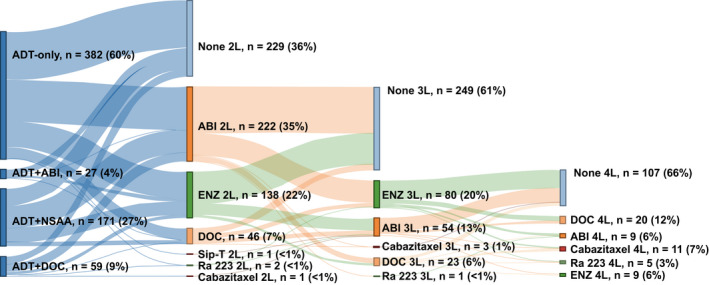
Proportion of patients with mCSPC who progressed to mCRPC and received life‐prolonging treatment in the 2L, 3L, and 4L settings. Patients in 3L and 4L assigned to "None" also include patients who were still on 2L or 3L therapy, respectively. Number of patients who advanced to 2L treatment: 639; number of patients who advanced to 3L treatment: 410 (does not include patients who did not have a 2L treatment); number of patients who advanced to 4L treatment: 161 (does not include patients who did not have a 2L or 3L treatment)

Of the 1395 patients, 639 (46%) progressed to mCRPC during the study period. NHT was the most commonly prescribed 2L (abiraterone: 35%; enzalutamide: 22%) and 3L (abiraterone: 13%, enzalutamide: 20%) treatment. A total of 229 (36%) patients who progressed did not receive any 2L treatment. Of these 229 patients, 80 (35%) died while 149 (65%) survived at a median follow‐up of 8.1 months after the date of mCRPC. Few patients were prescribed radium‐223, sipuleucel‐T, or cabazitaxel after progressing to mCRPC (Figure 3).

## 4 DISCUSSION

The landscape of treatment options and recommendations for patients with mCSPC has shifted significantly in recent years, beginning with the survival benefit observed with early administration of docetaxel followed by several NHTs. However, clinical trial data and guidelines do not always reflect real‐world treatment patterns. This retrospective, observational study in the VHA setting demonstrated that management of the mCSPC population (from April 2014 to March 2018) was dominated by ADT‐only (63%) and ADT + NSAA (24%). A small portion of men with more advanced disease received ADT + docetaxel (8%) or ADT + abiraterone (5%). Patients prescribed ADT + docetaxel were, on average, younger. While the use of abiraterone and docetaxel increased over time, it nevertheless remained low by the conclusion of the study period (ADT + docetaxel: 3%–9%; ADT + abiraterone: 1%–15%). For context, data disclosure dates for ADT + docetaxel and ADT + abiraterone were mid‐2014 and mid‐2017, respectively. Upon progression to mCRPC, the most commonly prescribed pharmacotherapies were abiraterone and enzalutamide. On multivariable analysis, no significant differences were detected between ADT + NSAA in time to mCRPC and OS compared with patients prescribed ADT‐only.

Only a few real‐world outcomes studies have evaluated treatment patterns and survival outcomes in a large cohort of patients with mCSPC.^16^, ^17^, ^18^ In line with our study, preliminary results from Ke et al., using a similar study period (2015–2018), indicated that the most common 1L therapy for mCSPC was ADT‐only (47%), followed by ADT + docetaxel (8%), ADT + abiraterone (7%), and ADT + NSAA (5%), and that the use of newer therapies had increased yet remained comparatively low.^16^, ^17^ Flaig et al. found that approximately half of the patients enrolled in a US claims database between 2000 and 2013 received ADT‐only, although the study period predated widespread use of docetaxel and NHTs.^18^ In both studies, >30% of patients did not receive any pharmacotherapy for mCSPC. Excluding non‐treated patients in the two studies, the proportion of patients prescribed ADT‐only (69% and 74%, respectively) is similar to that of our study (66%).

Of note, Black patients were more likely to receive ADT‐only, even though the VHA is an equal access system. While data on treatment by race were not adjusted for potential differences in baseline characteristics, these descriptive data do indicate less treatment intensification among Black patients and therefore disparity by race (Table S1). This is consistent with other studies showing lower rates of treatment intensification among Black patients, for example, a Medicare analysis (2004–2014) of patients with stage IV prostate cancer.^22^ However, more data over a longer time period with formal adjustment for key covariates are needed.

A substantial proportion (24%) of patients in our study received ADT + NSAA beyond the recommended duration for the treatment of testosterone flare.^4^ When correcting for available baseline characteristics, our study found no difference compared to ADT‐only in time to mCRPC or death, though unobserved differences in patient characteristics could have impacted results. Our study further supports the mixed opinion of the benefit of long‐term anti‐androgen therapy.^23^ A meta‐analysis reported that addition of an anti‐androgen (including cyproterone acetate) was associated with only a 2% improvement in 5‐year survival.^24^ An alternative interpretation of our data is that adoption of highly effective NHTs in the mCRPC setting in recent years may have contributed to the lack of survival difference with ADT + NSAA versus ADT alone.

Due to the low number of patients in the ADT + abiraterone and ADT + docetaxel cohorts, we were unable to evaluate the time to mCRPC and OS compared to ADT‐only by appropriately adjusting for patient characteristics. ADT + abiraterone and ADT + docetaxel were prescribed to patients with greater disease severity, suggesting that more intensive therapy is reserved for patients with more aggressive disease. However, between 2017 and 2018, even among those with visceral metastases, >40% received ADT alone. In the CHAARTED, STAMPEDE, and LATITUDE studies, adding docetaxel or abiraterone to ADT demonstrated consistent and large OS gains.^5^, ^6^, ^7^, ^8^, ^9^, ^10^, ^11^

In addition, IPTW‐adjusted median time to mCRPC was ~22 months and 3‐year OS was around 60% with ADT‐only and ADT + NSAA in our study. This is similar to the median PFS (range: 14.8–22.1 months) and 3‐year OS (range: 49%–61%) for the placebo + ADT arms of ARCHES (OS data not yet available), LATITUDE, STAMPEDE, and TITAN, as well as to the corresponding values for the ADT + NSAA arm (~24 months and 72%) in ENZAMET, but inferior to outcomes in the intensified treatment arms (NHT or docetaxel plus ADT) in all these trials.^7^, ^10^, ^12^, ^13^, ^14^, ^15^ Since the use of docetaxel and NHTs was low in the current study––despite increasing over time––our study suggests significant room for survival improvement in the mCSPC population.

The majority of the patients in our study who progressed to mCRPC received NHT (57%) as 2L therapy, while 7% received docetaxel. Slightly more than one‐third (36%), however, did not receive any 2L treatment after progressing to mCRPC. Our results are in line with George et al. who reported that, among 2559 commercially insured patients initiating mCRPC treatment between 2013 and 2017, the majority received NHTs (65%) as initial treatment, 15% underwent chemotherapy, and 23% did not receive any treatment.^25^ Similarly, in a recent VHA study of mCSPC patients treated with ADT + docetaxel or ADT + abiraterone between 2014 and 2018 who progressed to mCRPC, the vast majority were first treated with an NHT. Among those who received 2L treatment, NHT was most commonly prescribed.^26^

Limitations of the study include those inherent to claims database analyses, for example, coding errors, inability to guarantee actual treatment administration, and a potential lack of generalizability.^27^ The VHA is however the largest integrated health system in the United States, and any biases due to financial issues are less relevant in this patient population given equal access to care.^28^, ^29^ Importantly, these claims data do not capture information on prognostic factors such as performance status, Gleason score, number of metastases, and high versus low volume disease. In addition, the potential exists for unforeseen confounding of the comparison between ADT + NSAA versus ADT‐only.

## 5 CONCLUSIONS

Practice patterns for mCSPC in the VHA were largely dominated by use of ADT‐only treatment and ADT + NSAA, mostly bicalutamide, even in the most recent time period. Patients treated with ADT + NSAA experienced similar time to mCRPC and OS compared with ADT‐only, but results should be interpreted with caution as we could not adjust for all prognostic factors. Only a small segment of patients with higher risk disease received ADT + abiraterone or ADT + docetaxel. With the emergence of several newly approved NHTs associated with improved survival in a broad mCSPC population, our study suggests significant room for improved outcomes in this population. The results of this study may help to enhance our understanding of treatment patterns, disease progression, and survival rates of patients with mCSPC beyond the confines of a well‐controlled and restricted clinical trial environment.

## CONFLICT OF INTEREST

Stephen J. Freedland–Consulting or advisory role with Astellas Pharma, AstraZeneca, Sanofi, Bayer, Exact Sciences, Janssen, Pfizer, Dendreon, Sanofi, Merck, AstraZeneca, Myovant, and Clovis Oncology. Rickard Sandin, Birol Emir, and Lucile Serfass–Employees of Pfizer Inc., a study sponsor. Janvi Sah, Qiao Mu, and Anna Ratiu–Employed by SIMR, Inc., a paid consultant to the sponsors in connection with the development of this manuscript. Agnes Hong–Employee of Astellas Pharma Inc., a study sponsor. Scott T. Tagawa–Consulting or advisory role with Sanofi, Medivation/Astellas, Dendreon, Janssen, Genentech, Bayer, Endocyte, Eisai, Immunomedics, Karyopharm, Abbvie, Tolmar, Seattle Genetics, Amgen, Clovis, QED, Pfizer, AAA/Novartis, Clarity, Genomic Health, POINT Biopharma, Blue Earth Diagnostics, Aikido Pharma, and Gilead Sciences; research funding paid to his institution from Sanofi, Medivation, Astellas, Janssen, Amgen, Progenics, Dendreon, Lilly, Genentech, Newlink, BMS, Inovio, AstraZeneca, Immunomedics, Aveo, Rexahn, Atlab, Boehringer Ingelheim, Millennium, Bayer, Merck, Abbvie, Karyopharm, Endocyte, Clovis, Seattle Genetics, AAA/Novartis, POINT Biopharma, and Gilead Sciences.

## AUTHOR CONTRIBUTIONS

Stephen J. Freedland–Conceptualization, formal analysis, investigation, methodology, visualization, writing–original draft, writing–review and editing; Rickard Sandin–Conceptualization, formal analysis, funding acquisition, investigation, methodology, project administration, supervision, visualization, writing–original draft, writing–review and editing; Birol Emir–Conceptualization, formal analysis, investigation, methodology, visualization, writing–original draft, writing–review and editing; Lucile Serfass–Conceptualization, investigation, methodology, visualization, writing–original draft, writing–review and editing; Janvi Sah, Qiao Mu, and Anna Ratiu–Conceptualization, data curation, formal analysis, investigation, methodology, resources, software, validation, visualization, writing–original draft, writing–review and editing; Agnes Hong–Conceptualization, investigation, methodology, visualization, writing–original draft, writing–review and editing; Scott T. Tagawa–Conceptualization, formal analysis, investigation, methodology, visualization, writing–original draft, writing–review and editing.

## ETHICAL APPROVAL STATEMENT

The study was exempt from institutional review board or ethics committee approval as it utilized only de‐identified claims data.

## Supporting information

Fig S1

Table S1‐2

## Data Availability

Upon request, and subject to review, Pfizer will provide the data that support the findings of this study. Subject to certain criteria, conditions, and exceptions, Pfizer may also provide access to the related individual anonymized participant data. See https://www.pfizer.com/science/clinical‐trials/trial‐data‐and‐results for more information.
