# Supplementary figures and images for: Treatment patterns and survival in metastatic castration‐sensitive prostate cancer in the US Veterans Health Administration
Source: Cancer Med. 2021 Nov 2;10(23):8570–80. doi: 10.1002/cam4.4372 (PMC8633245; doi:10.1002/cam4.4372)

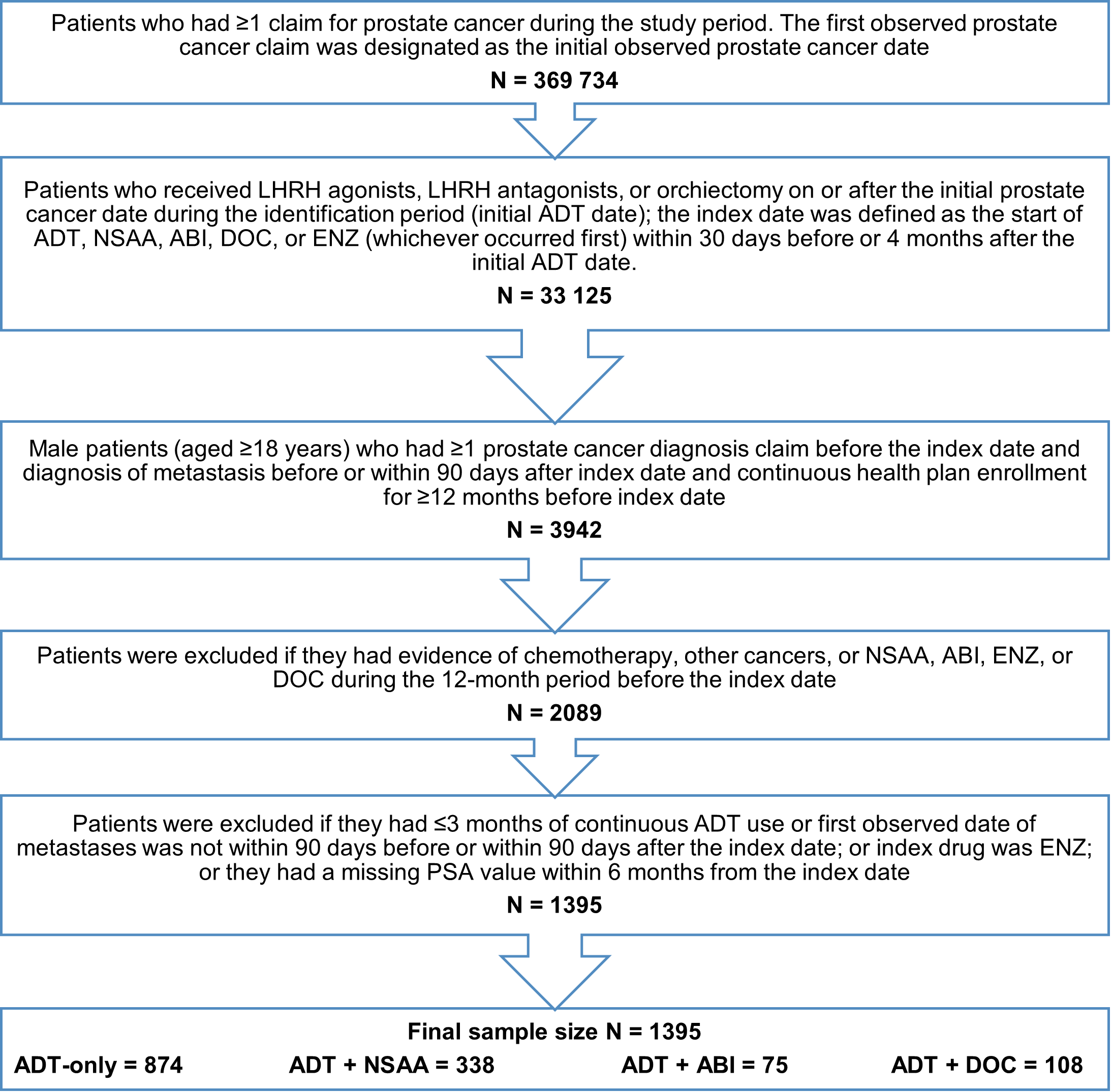

Supplement: Supplementary file 1 — Fig S1 [file CAM4-10-8570-s001.tif]
