# Supplementary material for: Treatment patterns and survival in metastatic castration‐sensitive prostate cancer in the US Veterans Health Administration
Source: Cancer Med. 2021 Nov 2;10(23):8570–80. doi: 10.1002/cam4.4372 (PMC8633245; doi:10.1002/cam4.4372)
Supplement: Supplementary file 2 — Table S1‐2 [file CAM4-10-8570-s002.docx]

Supporting Table 1. Treatment received by patients with mCSPC, by race – unadjusted, pre-IPTW

|  | **Race – White** | **Race – Black** | **Race – Other/unknown** |
| --- | --- | --- | --- |
| Sample size, n | 920 | 365 | 110 |
| ADT-only, n (%) | 555 (60.3) | 247 (67.7) | 72 (65.5) |
| ADT+NSAA, n (%) | 238 (25.9) | 77 (21.1) | 23 (20.9) |
| ADT+abiraterone, n (%) | 49 (5.3) | 18 (4.9) | 8 (7.3) |
| ADT+docetaxel, n (%) | 78 (8.5) | 23 (6.3) | 7 (6.4) |

Abbreviations: ADT, androgen deprivation therapy; IPTW, inverse probability treatment weighting; mCSPC, metastatic castration-sensitive prostate cancer; NSAA, nonsteroidal anti-androgen.

Supporting Table 2. Baseline demographic and clinical characteristics among patients with mCSPC – post-IPTW and post-PSM adjustment

|  | **Main analysis – IPTW** | | | **Sensitivity analysis – PSM** | | |
| --- | --- | --- | --- | --- | --- | --- |
|  | **ADT-only (reference)** | **ADT+NSAA** | **STD^a^** | **ADT-only (reference)** | **ADT+NSAA** | **STD^a^** |
| Sample size | 874 | 338 |  | 370 | 185 |  |
| Age (years) |  |  |  |  |  |  |
| Mean (SD) | 73.7 (9.9) | 73.7 (9.8) | 0.36 | 75.1 (8.9) | 75.1 (8.9) | N/A |
| Median (Q25–Q75) | 72 (67–82) | 73 (67–82) |  | 73 (68–82) | 73 (68–82) |  |
| Age (y), n (%) |  |  |  |  |  |  |
| ≤59 | 58 (7) | 22 (7) | 0.55 | 8 (2) | 4 (2) | N/A |
| 60–69 | 291 (33) | 110 (33) | 1.49 | 120 (32) | 60 (32) | N/A |
| 70–79 | 248 (28) | 94 (28) | 1.43 | 116 (31) | 58 (31) | N/A |
| ≥80 | 276 (32) | 112 (33) | 3.17 | 126 (34) | 63 (34) | N/A |
| Race, n (%) |  |  |  |  |  |  |
| White | 571 (65) | 216 (64) | 2.76 | 264 (71) | 132 (71) | N/A |
| Black | 234 (27) | 94 (28) | 2.16 | 82 (22) | 41 (22) | N/A |
| Other/unknown | 69 (8) | 28 (8) | 1.30 | 24 (6) | 12 (6) | N/A |
| Treatment year, n (%) |  |  |  |  |  |  |
| 2014 (Q2–Q4) | 223 (26) | 85 (25) | 0.87 | 95 (26) | 53 (29) | 6.67 |
| 2015 | 229 (26) | 89 (26) | 0.41 | 86 (23) | 48 (26) | 6.27 |
| 2016 | 207 (24) | 81 (24) | 0.74 | 95 (26) | 43 (23) | 5.65 |
| 2017–Q1 2018 | 215 (25) | 83 (24) | 0.27 | 94 (25) | 41 (22) | 7.61 |
| Pre-index treatment, n (%) |  |  |  |  |  |  |
| ADT (LHRH agonist/antagonist) | 28 (3) | 10 (3) | 0.51 | 10 (3) | 4 (2) | 3.50 |
| Radiation therapy | 67 (8) | 23 (7) | 2.93 | 27 (7) | 14 (8) | 1.03 |
| Chronic corticosteroid use | 56 (6) | 23 (7) | 1.37 | 11 (3) | 4 (2) | 5.12 |
| NCI Comorbidity Index score |  |  |  |  |  |  |
| Mean (SD) | 1.5 (1.7) | 1.5 (1.7) | 1.33 | 1.4 (1.7) | 1.5 (1.8) | 6.72 |
| Median (Q25–Q75) | 1 (0–2) | 1 (0–2) |  | 1 (0–2) | 1 (0–2) |  |
| Baseline comorbidities, n (%) |  |  |  |  |  |  |
| Acute coronary syndrome | 56 (6) | 22 (6) | 0.23 | 19 (5) | 12 (6) | 5.77 |
| Angina pectoris | 22 (2) | 9 (3) | 1.66 | 5 (1) | 5 (3) | 9.58 |
| Arrhythmia | 70 (8) | 27 (8) | 0.01 | 25 (7) | 14 (8) | 3.14 |
| Chronic obstructive pulmonary disease | 138 (16) | 54 (16) | 0.38 | 51 (14) | 31 (17) | 8.25 |
| Congestive heart failure | 77 (9) | 32 (10) | 2.57 | 28 (8) | 15 (8) | 2.01 |
| Diabetes | 282 (32) | 106 (31) | 2.03 | 114 (31) | 65 (35) | 9.19 |
| Hyperlipidemia | 472 (54) | 182 (54) | 0.12 | 200 (54) | 100 (54) | 0.00 |
| Hypertension | 641 (73) | 247 (73) | 0.66 | 272 (74) | 132 (71) | 4.83 |
| Myocardial infarction | 31 (4) | 13 (4) | 1.51 | 9 (2) | 4 (2) | 1.80 |
| Stroke | 55 (6) | 21 (6) | 0.76 | 23 (6) | 12 (6) | 1.11 |
| Urinary tract infection | 93 (11) | 34 (10) | 2.38 | 36 (10) | 17 (9) | 1.84 |
| Obesity | 283 (32) | 109 (32) | 0.46 | 119 (32) | 57 (31) | 2.90 |
| Prognostic variables^b^ |  |  |  |  |  |  |
| PSA (ng/mL) |  |  |  |  |  |  |
| Mean (SD) | 280.4 (732.6) | 266.2 (730.3) | 1.94 | 272.7 (700.0) | 198.7 (506.4) | 12.11 |
| Median (Q25-Q75) | 35 (9-141) | 39 (11-148) |  | 36 (10-142) | 39 (10-148) |  |
| Hemoglobin value (G/DL) |  |  |  |  |  |  |
| Mean (SD) | 12.9 (2.1) | 12.9 (2.0) | 0.47 | 13.0 (2.0) | 13.0 (2.0) | 2.76 |
| Median (Q25–Q75) | 13 (12–14) | 13 (12–14) |  | 13 (12–14) | 13 (12–14) |  |
| Alkaline phosphatase value (IU/L) |  |  |  |  |  |  |
| Mean (SD) | 206.9 (275.7) | 209.3 (283.2) | 0.86 | 199.3 (242.1) | 218.7 (291.1) | 7.26 |
| Median (Q25–Q75) | 105 (75–257) | 104 (75–255) |  | 111 (75–274) | 105 (76–276) |  |
| Time from metastatic diagnosis to index date (days)^c^ |  |  |  |  |  |  |
| Mean (SD) | 2.8 (36.9) | -8.8 (33.9) | 32.78 | 2.2 (37.2) | -7.7 (33.6) | 27.98 |
| Median (Q25–Q75) | 6 (-13–23) | 1 (-27–8) |  | 5 (-16–22) | 1 (-24–9) |  |
| Site of metastasis,^c^ n (%) |  |  |  |  |  |  |
| Lymph nodes | 194 (22) | 74 (22) | 0.86 | 70 (19) | 31 (17) | 5.64 |
| Respiratory and digestive | 49 (6) | 20 (6) | 0.55 | 22 (6) | 9 (5) | 4.77 |
| Other sites (including bone) | 665 (76) | 263 (78) | 4.14 | 289 (78) | 148 (80) | 4.64 |
| Unspecified | 123 (14) | 39 (11) | 7.85 | 55 (15) | 21 (11) | 10.40 |

Abbreviations: ADT, androgen deprivation therapy; IPTW, inverse probability treatment weighting; LHRH, luteinizing hormone-releasing hormone; mCSPC, metastatic castration-sensitive prostate cancer; N/A, not applicable; NCI, National Cancer Institute; NSAA, nonsteroidal anti-androgen; PSM, propensity score matching; PSA, prostate-specific antigen; Q25-Q75, interquartile range; SD, standard deviation; STD, standardized difference.

^a^STD: standardized difference=100* (actual STD) measured versus ADT-only. A standardized difference greater than 10 is considered significant. Age and race were hard-matched, precluding STD calculations for these variables.

^b^Prognostic variables were evaluated within 6 months prior to the index date.

^c^Metastatic related variables were evaluated within ± 90 days from the index date.
